# The Role of Stem Cell Factor in Hyperpigmented Skin Lesions

**DOI:** 10.31557/APJCP.2019.20.12.3723

**Published:** 2019

**Authors:** Aliaa Atef, Mohamed A El-Rashidy, Amal Abdel Azeem, Ahmed M Kabel

**Affiliations:** 1 *Department of Pathology, *; 2 *Department of Dermatology and Venereology, *; 3 *Department of Pharmacology, Faculty of Medicine, Tanta University, Tanta, Egypt, *; 4 *Department of Clinical Pharmacy, College of Pharmacy, Taif University, Taif, Saudi Arabia. *

**Keywords:** Stem cell factor, melisma, basal cell carcinoma, melanoma

## Abstract

**Background:**

Skin hyperpigmentation usually results from an increased number, or activity, of melanocytes. The degree of pigmentation of skin depends on the amount and type of melanin, degree of skin vascularity, presence of carotene, and thickness of the stratum corneum. Common causes of hyperpigmentation include post-inflammatory hyperpigmentation, melasma, solar lentigines, ephelides (freckles), and café-au-lait macules. Some skin tumors can be hyperpigmented as basal cell carcinoma (BCC), squamous cell carcinoma (SCC) and malignant melanoma (MM). Stem cell factor (SCF) is a growth factor and its interaction with its receptor, c-kit, is well known to be critical to the survival of melanocytes.

**Methods:**

This study was carried out on 60 patients complaining of hyperpigmented skin lesions (20 melasma, 20 solar lentigines, and 20 freckles) and 36 patients with skin tumors (14 BCC, 12 SCC, and 10 MM). Punch skin biopsies were taken from the previous lesions. Immunohistochemical staining of these samples was done using the stem cell factor (SCF).

**Results:**

There was positive expression of SCF in all cases of melasma, solar lentigines and freckles with significant increase in the intensity of expression in the lesional areas than the non-lesional ones (P=0.004). There was also a statistically significant increase in the expression of SCF in BCC and melanoma tumor cells.

**Conclusion:**

SCF has a great role in skin hyperpigmented disorders and this can be used as a target for the developing of new antipigmentary lines of treatment by inhibiting SCF. SCF can also be involved in the emergence of some skin tumors.

## Introduction

Skin color is one of the most particular ways in which humans differ and was globally used to define various human races. Skin pigmentation depends on the amount and type of melanin, degree of the vascular supply, the presence of carotene, and the thickness of the stratum corneum (Kabel et al., 2016). Skin hyperpigmentation results from the increased number or activity of melanocytes. Hyperpigmentation can be represented in various forms including post-inflammatory hyperpigmentation, melasma, ephelides (freckles), solar lentigines, and café-au-lait macules (Plensdorf and Martinez, 2009). Interestingly, hyperpigmentation could be seen in some skin tumors such as basal cell carcinoma (BCC), squamous cell carcinoma (SCC) and malignant melanoma (MM) (McCormack et al., 2013; Kabel et al., 2016).

Ephelides or freckles are multiple small, pale, brown macular lesions with ill-defined margins. They can be more encountered in summer, located in sun-exposed areas of fair skin (Bastiaens et al., 2004). As for solar lentigines, they are common aging spots with increased epidermal pigmentation and considered the hallmark of aged skin (Kadono et al., 2001). Melasma, as well, is another hyperpigmented skin disorder characterized by symmetric brown to grey macules and patches with serrated and geographic outlines (Bandyopadhyay, 2009; Altaei, 2012).

Human keratinocytes secrete different types of cytokines in response to various stimuli that act as mitogens or melanogens for human melanocytes. These cytokines include melanocyte-stimulating hormone, endothelin-1, fibroblast growth factor and Stem cell factor (SCF). Human fibroblasts also secrete several melanogenic cytokines as SCF and hepatocyte growth factor, which suggests that over-expression of these cytokines by dermal fibroblasts may activate melanocytes in the overlying epidermis (Yoshida et al., 2001; Imokawa, 2004).

Stem cell factor is a growth factor that binds to the receptor tyrosine kinase (RTK) and leads to activation of multiple signal transduction factors. SCF has a role in the differentiation, survival and self-renewal of hematopoietic stem cells (Roskoski, 2005). The interaction of stem cell factor with its receptor, c-kit, is well known to be crucial for the survival of melanocytes. Little is known about the role of SCF/c-kit interaction in epidermal pigmentation (Kim et al., 2018). SCF has been implicated in many disease processes characterized by tissue remodelling and fibrosis through its effect on the activities of mast cells which are involved in inflammatory skin disorders and wound healing processes. This can be explained by the role of SCF in mast cell survival, growth, migration, and activation. SCF induces differentiation of mast cells from pluripotent progenitor cells in human bone marrow or peripheral blood. SCF also suppresses mast cell apoptosis and stimulates mast cell adhesion to extracellular matrix proteins. At the same time, SCF activates mast cells for secretion of mediators such as tumor necrosis factor-α, prostaglandin E, glycosaminoglycans and proteolytic enzymes (Vliagoftis et al., 1997; Huttunen et al., 2002).

*There are two main forms*

Soluble and membrane-bound forms. The soluble form secreted by dermal fibroblasts stimulates epidermal melanocytes by the dispersion pathway through the basement membrane from the dermis towards the epidermis It regulates melanogenesis under homeostatic, stimulatory or pathogenic conditions. SCF/KIT signaling also mediates ultraviolet B-induced pigmentation and several pigmentation disorders (Kang et al., 2006). 

On the other hand, SCF can be also expressed in basal, squamous cell carcinoma and malignant melanoma tumor islands. It is thought to be produced by the tumor cells and may account for the increased number of stromal mast cells which induce fibroplasia of the surrounding stroma (Yamamoto et al., 1997).

Although most pigmentation disorders are benign or nonspecific, some disorders of skin pigmentation present cosmetic or psychological challenges to the patient, necessitating evaluation and treatment. Others may be indicators of underlying systemic disease or primary skin malignancy. Proper diagnosis of these common skin conditions will allow the physician to facilitate appropriate skin treatment and reassure the patient (Kang et al., 2006). Therefore, this study intended to investigate the expression of SCF in some of the pigmented skin lesions to determine its role in the pathogenesis and usefulness in therapy.

## Materials and Methods

*Experimental design*

This study was carried out on 60 patients with different types of epidermal hyperpigmentation and 36 patients with pigmented skin tumors (14 BCC, 12 SCC, and 10 MM). They were 74 females and 22 males. They included 20 patients with melasma, 20 patients with solar lentigines, 20 patients with freckles. Skin tumors group included 14 patients with basal cell carcinoma, 12 patients with squamous cell carcinoma and 10 patients with malignant melanoma. A written consent was taken from all patients before being involved in this study. All patients were subjected to complete history taking and thorough general and dermatological examinations. Punch biopsy (3.5-5 mm) was taken from the nearby normal skin beside the lesion to serve as a control. The punch biopsies taken were for experimental purpose and informed consents were signed by the patients of the study. All specimens were preserved immediately in 10% neutral buffered formalin solution for 24-48 hours, processed and embedded in paraffin blocks. 

*Immunohistochemistry*

Immunohistochemical staining for stem cell factor (*SCF*) was done to evaluate its expression in different studied lesions. Immunohistochemistry was performed using the immunoperoxidase method on 4-m-thick sections from formalin-fixed, paraffin-embedded blocks. The antigen retrieval (PBS buffer; pH 7.4) was done for all sections and were incubated with the primary antibody stem cell factor (mouse monoclonal IgG antihuman antibody (Santa Cruz Biotechnology Inc.) for 2 hours at room temperature. The sections were incubated with secondary antibody for 15 min at room temperature. 

Cytoplasmic staining was considered positive for *SCF* expression. Tissue was scored according to Hattori et al., (2004) based on the intensity of immunostaining in the positive cells (0%) = negative (0), weak (+1), moderate (+2) and strong (+3) (Hattori et al., 2004). Immunohistochemical staining was evaluated independently by two pathologists.

*Statistical analysis*

Statistical analysis was performed by using Associations between two categorical variables were done by two samples ‘t’ test/Mann Whitney/Analysis of variance/Kruskal-Wallis test. P-values less than 0.05 were considered statistically significant.

## Results

This study included 60 patients with hyperpigmented skin lesions diagnosed clinically and histopathologically confirmed as melasma, solar lentigines and freckles, and 36 patients with skin tumors which were divided into 14 BCC, 12 SCC, and 10 MMs.

The age of patients with melasma ranged from 35 to 55. Patients with solar lentigines aged between 45 and 70 and freckles patients from 25 to 65 years. Patients with skin tumors aged between 45 and 70 years. Concerning family history, 6 melasma cases (30%), 12 lentigines cases (60%) and 16 freckles cases (80%) showed positive family history. BCC cases showed one case (7%) with positive family, melanoma cases showed 4 cases (40%) with family history and SCC cases showed no family history. Three cases of BCC (21.4%) and 2 cases of SCC (16.7%) and one case of melanoma (10%) reported personal history of previous incidence in the same individual. All patients had lesions in the sun-exposed areas.

**Table 1:** The Immunohistochemical Staining Results of SCF in Studied Melasma, Lentigines and Freckles Cases

Intensity	Lesional		Perilesional		P-value
	No	%	No	%	
Melasma					0.004*
0	-	-	-	-	
+1	-	-	20	100	
+2	12	60	-	-	
+3	8	40	-	-	
Lentigines					0.004*
0	-	-	4	20	
+1	2	10	16	80	
+2	10	50	-	-	
+3	8	40	-	-	
Freckles					0.004*
0	-	-	4	20	
+1	4	20	14	70	
+2	6	30	2	10	
+3	10	50	-	-	

* P-value showed significant difference in the intensity of SCF expression (p-value < 0.05).

**Figure 1:**
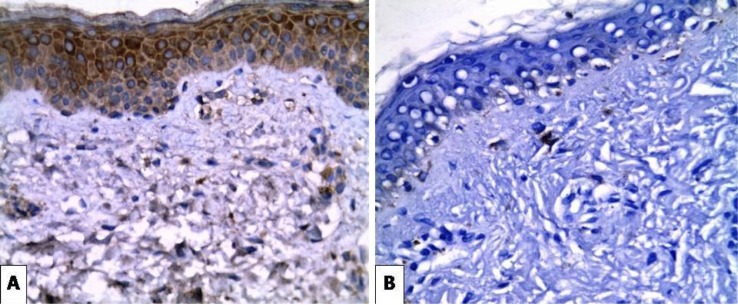
(A) Strong Positive Expression of SCF in Epidermal Keratinocytes and Dermal Fibroblasts of Melasma Case (X400). (B) Negative SCF in the Perilesional Area of the Same Case (X400).

**Figure 2:**
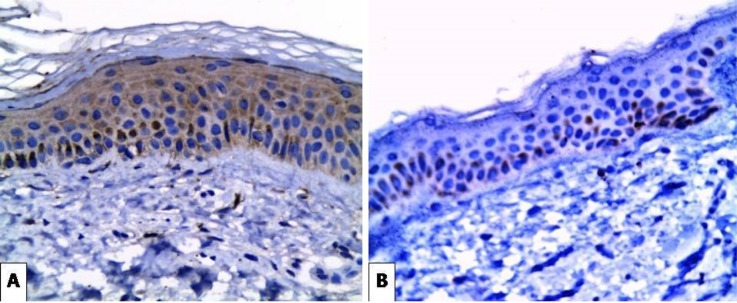
(A) Moderate Cytoplasmic Expression of SCF in Epidermal Keratinocytes and Dermal Fibroblasts of Solar Lentigines Case (X400). (B) Very Weak Basal Expression of SCF in the Perilesional Area of the Same Case (X400)

**Figure 3:**
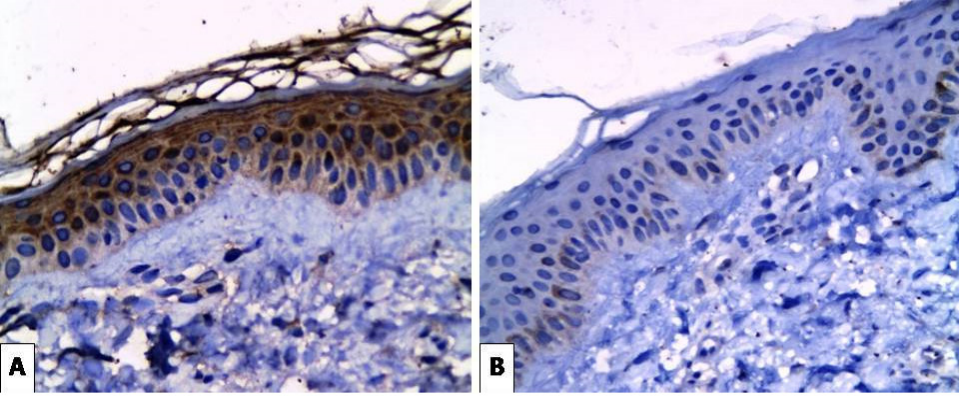
(A) Strong Expression of SCF in Epidermal Keratinocytes and Dermal Fibroblasts of Freckles Case (X400). (B) Very Weak Basal Expression of SCF in the Perilesional Area of the Same Case (X400)

**Figure 4:**
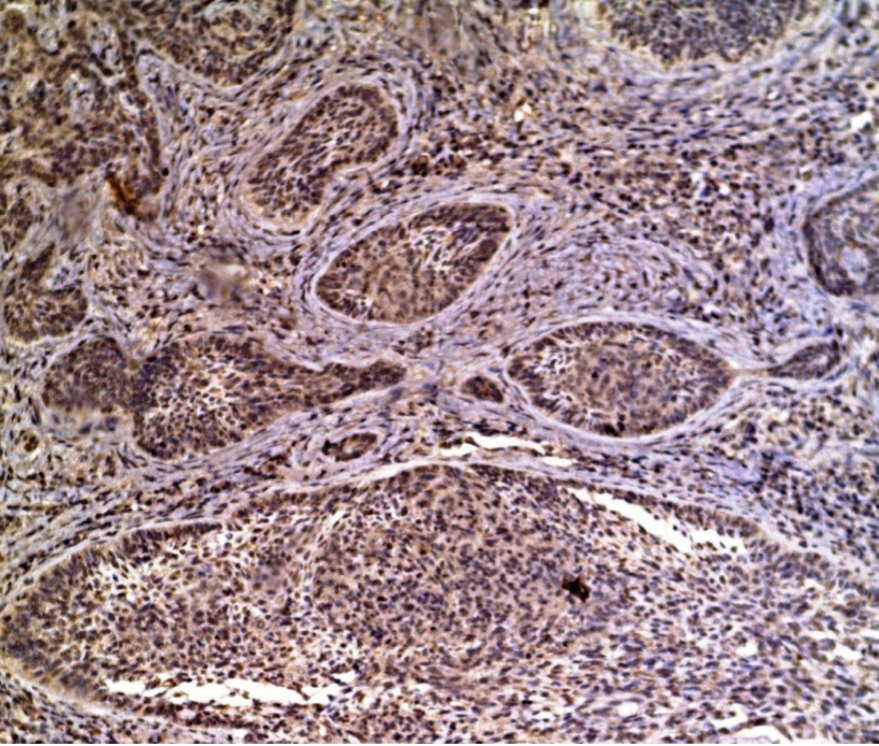
BCC Showing Strong Expression of SCF in Malignant Tumor Cells (X100).

**Table 2:** The Immunohistochemical Staining Results of SCF in Studied Skin Tumors

Intensity	Lesional		Perilesional		P-value
	No	%	No	%	
BCC					0.0124*
0	4	28.6	9	64.3	
+1	4	28.6	5	35.7	
+2	2	14.3	-	-	
+3	4	28.5	-	-	
SCC					0.4467
0	6	50.0	5	41.7	
+1	4	33.4	3	25.0	
+2	1	8.3	4	33.3	
+3	1	8.3	-	-	
Melanoma					0.004*
0	-	-	2	20.0	
+1	1	10.0	8	80.0	
+2	5	50.0	-	-	
+3	4	40.0	-	-	

* P-value showed significant difference in the intensity of SCF expression (p-value < 0.05).

**Figure 5:**
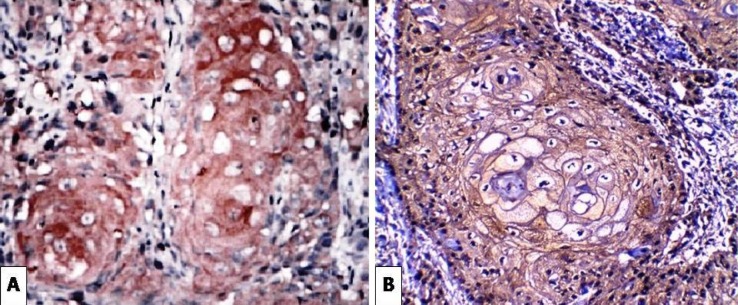
(A, B) Positive SCF Expression in Squamous Cell Carcinoma (X400)

**Figure 6:**
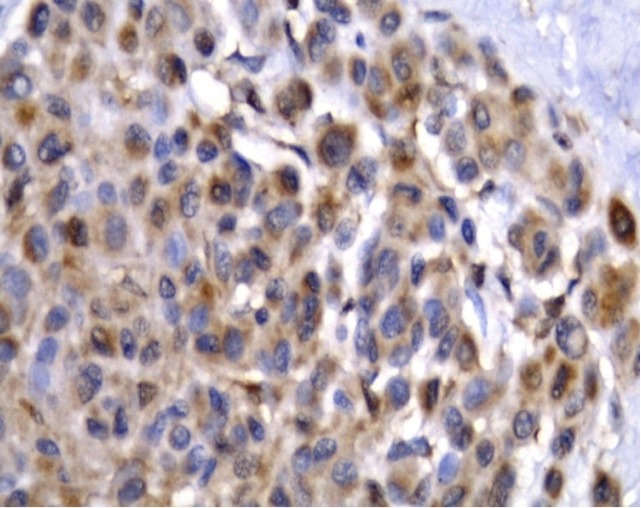
Malignant Melanoma Showing Positive SCF Expression in Malignant Tumor Cells (X400).

*Immunohistochemical staining results of SCF*

Positive expression of *SCF* was seen in all 20 cases of melasma. The intensity of SCF expression in melasma was increased in the lesional compared to the perilesional parts. There was moderate expression in (60%) of patients and (40%) strongly expressed in lesional areas, while in perilesional areas there was weak expression in (100%) of cases (Figure 1). Regarding the cases of lentigines, the intensity of *SCF* expression was also increased in the lesional parts with weak expression in (10%) of patients , moderately expressed in (30%) of patients and strongly expressed in (60%) of patients, while in the perilesional area there was no *SCF* expression in (20%) of patients and weak expression in (80%) of patients (Figure 2). The intensity of *SCF* expression in freckles cases was increased in the lesional parts. SCF was weakly expressed in (20%) of patients, moderately expressed in (30%) of patients and strongly expressed in (50%) of patients, while in the perilesional area, *SCF* was not expressed in (20%) of patients, weakly expressed in (70%) of patients and moderately expressed in (10%) of patients (Figure 3). Statistical analysis showed significant difference in the intensity of *SCF* expression between lesional and perilesional areas in the studied melasma, lentigines and freckles cases (Table 1).

BCC, SCC and MM cases showed cytoplasmic expression of *SCF* with variable intensities (Figure 4, 5, 6). There was a statistically significant increase in the expression in BCC and melanoma tumor cells (Table 2). 

## Discussion

Among the different hyperpigmented skin lesions, melasma, solar lentigines, and freckles are the most commonly encountered lesions. In addition, some malignant skin tumors as BCC, SCC, and MM can appear hyperpigmented. A paracrine linkage has been reported between keratinocytes, fibroblasts, and melanocytes. Several cytokines and chemokines such as SCF, α MSH, histamine, endothelin -1 and prostaglandins were found to be released from epidermal keratinocytes in response to ultraviolet B rays exposure (Kasamatsu et al., 2014).

*SCF* is a growth factor that is expressed by fibroblasts and endothelial cells throughout the body, promoting differentiation, proliferation, migration, and survival of melanocytes. The regulation of *SCF* expression on the gene level is a complicated process. Ultraviolet B light stimulates the expression of *SCF* in human epidermis. The mechanism of this *SCF* gene expression by ultraviolet B is unknown (Lennartsson and Ronnstrand, 2012).

In the present study, (30%) of melasma cases (60%) of lentigines cases and (80%) of freckles cases showed positive family history. Approving of these findings, Yang et al., (2008) suggested that familial factors were important in determining individual susceptibility to freckles development. Several other studies discussed the predisposing factors of these hyperpigmented lesions. Ezzedine et al., (2013) found that two factors are independently associated with the development of facial freckles, frequent sunburns and the presence of melanocortin receptor gene-1 with diminished function. Katoulis et al., (2010) also mentioned that genetic predisposition and chronic solar exposure can be important pathogenic precipitating factors in solar lentigines. Ortonne et al., (2009) as well, reported that family history of melasma plays a role in its development.

BCC cases in the present study showed one case (7%) with positive family, melanoma cases showed 4 cases (40%) with family history and SCC showed no family history. Three BCC cases, 2 SCC and one melanoma case declared previous personal history. High and Zedan (2005) discussed the occurrence of BCC as a part of an inherited genetic disorder called naevoid basal cell carcinoma syndrome. Interestingly, Asgari et al., (2015) stated that family history of skin cancer is an important independent risk factor for skin SCCs. In 1992, Karagas et al., (1992) found that persons with previous non-melanoma skin cancer had a 5-year risk of developing another tumor of the same histologic type. History of previous skin cancers, solar damage, and skin sensitivity to sun exposure were particularly related associated with higher risk. Firnhaber (2012) noted that personal history of skin cancer or the presence of first-degree relatives with melanoma can be risk factors for non-melanoma skin cancer in addition to malignant melanoma.

Our immunohistochemical results revealed that melasma cases showed significant increase in the intensity of SCF immunostaining in the lesional areas more than the perilesional normal ones (p<0.05). This was supported by the results of a study done by Kang et al., (2006). They suggested that SCF was expressed in melasma more than the surrounding normal skin. They also reported that there was a difference in SCF immunostaining between melasma lesional and perilesional dermal fibroblasts. 

Regarding our immunohistochemical SCF staining results in solar lentigines cases, there was also significant increased intensity of* SCF* expression in lesional than perilesional areas and dermal fibroblasts (p<0.05). Similarly, Kovacs et al., (2010) suggested that SCF was expressed in lentigines more than the perilesional normal skin. They highlighted the importance of the contribution of fibroblasts in regulating hyperpigmentation through keratinocyte growth factor and SCF. Besides, Hattori et al., (2004) reported that soluble SCF secreted by dermal fibroblasts plays an important role in activating epidermal melanocytes via a dispersion pathway through the basement membrane from the dermis toward the epidermis.

The studied cases of freckles in the present study also showed similar positive immunohistochemical staining results for SCF in the lesional parts. This can be attributed to the stimulatory effect of SCF as cytokine on the proliferation and activation of melanocytes. Shin et al., (2010) stated that repeated ultraviolet rays exposure stimulates fibroblasts to secrete SCF directly and indirectly (via keratinocytes). The increased secretion of SCF was probably associated with fibroblast senescence caused by ultraviolet rays which can be related to the pathogenesis of hyperpigmented lesions in chronically sun-damaged skin.

Studying the BCC cases showed significant increased intensity of *SCF* expression in tumor cells compared to normal surrounding keratinocytes. Similar results were discovered by Yamamoto et al., (1997). They found positive expression of *SCF* in the BCC tumor islands with the center of these islands being strongly positive. SCF was also detected on fibroblast-like cells and mast cells in the stroma. Their study also demonstrated that abundant SCF produced by the tumor cells may account for the increased number of stromal mast cells, which induce fibroplasia of the tumor stroma. 

The studied SCC cases showed 50% cytoplasmic positivity for *SCF* expression. In approval with our results, Satomura et al., (2007) revealed prominent expression of *SCF* in the malignant squamous cells of the pigmented SCC, while the non-pigmented SCC showed little expression. These findings strongly suggest that SCF secreted by malignant squamous cells which stimulates proliferation and differentiation of melanocytes is implicated in the pathogenesis of SCC. Interestingly, another study done by Perumal et al., (2014) reported that SCF as a major cytokine and the ligand for the c-Kit proto-oncogene was found to be overexpressed in human lung adenocarcinomas, but not in squamous cell carcinomas.

Malignant melanoma cases in our study showed significant positive expression of *SCF*. In 1992, Turner et al. found SCF receptors on the surfaces of melanoma cell lines. They suggested the possibility that autocrine production of SCF by c-kit receptor bearing tumor cells may enhance cell growth in tumor cell lines. In 1995, Papadimitriou et al. noted that all tested melanoma cases expressed *SCF* as assessed at mRNA level. On the contrary, Takahashi et al., (1995) observed positive *SCF* expression only in one case out of five melanoma cases. They also found that malignant melanoma showed less expression of* SCF* than in benign melanocytic tumors. Prignano et al., (2006) also noticed positive SCF staining but in a minority of metastatic melanoma cases. Additionally, Caslin et al., (2018) suggested that SCF partly causes the increased number of mast cells, which may enhance tumor cell proliferation, leading to tumor progression.

In conclusion, our study findings strongly suggest that SCF has a great role in the development of various benign and malignant skin hyperpigmented disorders. Further studies are yet needed to investigate the possibility of using SCF as a target for the developing of new antipigmentary lines or new targeted therapy for skin cancers by inhibiting SCF and for more clarification of the role of SCF in hyperpigmented benign and malignant skin lesions.
